# CRISPR/Cas9-mediated gene knockout screens and target identification via whole-genome sequencing uncover host genes required for picornavirus infection

**DOI:** 10.1074/jbc.M117.782425

**Published:** 2017-04-26

**Authors:** Heon Seok Kim, Kyungjin Lee, Sangsu Bae, Jeongbin Park, Chong-Kyo Lee, Meehyein Kim, Eunji Kim, Minju Kim, Seokjoong Kim, Chonsaeng Kim, Jin-Soo Kim

**Affiliations:** From the ‡ Center for Genome Engineering, Institute for Basic Science, Seoul 151-747, South Korea,; the § Department of Chemistry, Seoul National University, Seoul 151-747, South Korea,; the ¶ Center for Convergent Research of Emerging Virus Infection and; ** Virus Research and Testing Center, Korea Research Institute of Chemical Technology, Daejeon 34114, South Korea,; the ‖ Department of Chemistry, Hanyang University, Seoul 04763, South Korea, and; ‡‡ ToolGen, Inc., Byucksan Kyoungin Digital Valley 2-Cha, Geumcheon-Gu, Seoul 153-023, South Korea

**Keywords:** CRISPR/Cas, host-pathogen interaction, poliovirus, RNA virus, sialic acid, CRISPR screen, Enterovirus D68, whole-genome sequencing

## Abstract

Several groups have used genome-wide libraries of lentiviruses encoding small guide RNAs (sgRNAs) for genetic screens. In most cases, sgRNA expression cassettes are integrated into cells by using lentiviruses, and target genes are statistically estimated by the readout of sgRNA sequences after targeted sequencing. We present a new virus-free method for human gene knockout screens using a genome-wide library of CRISPR/Cas9 sgRNAs based on plasmids and target gene identification via whole-genome sequencing (WGS) confirmation of authentic mutations rather than statistical estimation through targeted amplicon sequencing. We used 30,840 pairs of individually synthesized oligonucleotides to construct the genome-scale sgRNA library, collectively targeting 10,280 human genes (*i.e.* three sgRNAs per gene). These plasmid libraries were co-transfected with a Cas9-expression plasmid into human cells, which were then treated with cytotoxic drugs or viruses. Only cells lacking key factors essential for cytotoxic drug metabolism or viral infection were able to survive. Genomic DNA isolated from cells that survived these challenges was subjected to WGS to directly identify CRISPR/Cas9-mediated causal mutations essential for cell survival. With this approach, we were able to identify known and novel genes essential for viral infection in human cells. We propose that genome-wide sgRNA screens based on plasmids coupled with WGS are powerful tools for forward genetics studies and drug target discovery.

## Introduction

Genome-scale libraries of transcriptional activator-like effector nucleases (1, 2) and RNA-guided endonucleases (3–8), which consist of the Cas9 protein and small guide RNAs (sgRNAs),^4^ originated from the type II clustered regularly interspaced repeat (CRISPR)-CRISPR-associated (Cas) prokaryotic adaptive immune system, are now available for forward genetic screens in human and other mammalian cells. Several groups have constructed genome-wide libraries of lentiviruses encoding sgRNAs by cloning oligonucleotides synthesized *in situ* on a microarray and used them to search for genes whose disruption in human and murine cells gives rise to oncogenesis (3), drug resistance (4–7), viral replication (8), etc. In these systems, target genes are identified indirectly by comparing the number of each sgRNA sequence before and after selection via high-throughput sequencing. In this study, we sought to develop a new method for genome-wide knockout screens using sgRNA-encoding plasmids rather than lentiviruses and target gene identification via WGS rather than targeted amplicon sequencing.

Here we chose two human pathogenic viruses, poliovirus (PV) and enterovirus D68 (EV-D68), which are members of the Picornaviridae family, which causes a wide range of illnesses such as poliomyelitis and childhood respiratory diseases (9, 10). In 2014, outbreaks in the United States of severe respiratory illness caused by EV-D68 raised concerns regarding this emerging pathogen (11). These viruses are non-enveloped, single-stranded, positive-sense RNA viruses. Currently, there are no approved therapies.

Infection by these viruses is a complex process that includes many steps, such as receptor binding, viral entry, translation, replication, and viral release. Each step requires host factors, and interactions between host and viral factors are essential to fulfill successful infection. Identification of these host factors could help to elucidate novel molecular mechanisms of viral infection and facilitate the development of novel targets for antiviral drugs.

These viruses are highly cytopathic, so infected host cells are eventually killed. Only cells lacking key host factors essential for viral infection can survive. The CRISPR system induces permanent knockout of the target gene. By combining these two features, it is possible to screen key host factors for virus infection by selection of survived cells after virus challenge and target identification.

## Results

### Gene knockout screens using pooled sgRNA libraries

A total of 30,840 pairs of oligonucleotides that encoded sgRNAs, collectively targeting 10,280 protein-coding genes, were individually synthesized and cloned in an sgRNA expression plasmid. Using Cas-OFFinder and Cas-Designer (available at http://www.rgenome.net),^5^ we carefully chose three target sites per gene to avoid off-target effects and in-frame mutations as much as possible (12, 13). All sgRNAs in the library contained two extra guanine nucleotides to produce ggX_20_ sgRNAs, further reducing off-target effects (14, 15).

**Figure 1.**
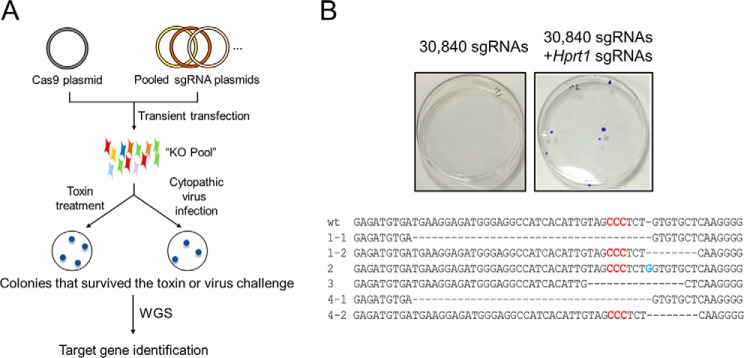
**Gene knockout screens using pooled sgRNA libraries.** *A*, schematic of forward genetic screens in human cells using pooled sgRNA libraries. *B*, crystal violet staining of 6-TG-resistant cells transfected previously with the sgRNA library. 6-TG-resistant cells appeared when sgRNAs targeting *HPRT1* were diluted in the library containing 30,840 sgRNAs. The mutated sequences from surviving clones are shown below. *Red letters* indicate the PAM, and the *blue letter* indicates an insertion.

To test whether cells resistant to viral infection or drug treatment could be selected by transient transfection of pooled sgRNA plasmids (Fig. 1*A* and supplemental Table S1), we mixed three sgRNA plasmids specific to the hypoxanthine-guanine phosphoribosyltransferase (*HPRT1*) gene with the library of 30,840 sgRNA plasmids and co-transfected this mixture into HeLa cells with the Cas9 plasmid. Transfected cells were treated with 6-thioguanine (6-TG), which is cytotoxic to cells that express the *HPRT1* gene. Several colonies survived 6-TG treatment (Fig. 1*B*), showing that the *HPRT1* gene was disrupted efficiently in HeLa cells. Sanger sequencing showed that each resistant colony had mutations at sgRNA target sites in the *HPRT1* gene, leading to the gene knockout. As expected, no colonies were obtained from cells transfected with the library alone (Fig. 1*B*). These results show that an sgRNA plasmid in a pool of tens of thousands of sgRNAs can still direct Cas9 to induce complete knockout of a target gene in human cell lines.

### Pooled sgRNA screens for poliovirus and enterovirus D68 resistance

**Figure 2.**
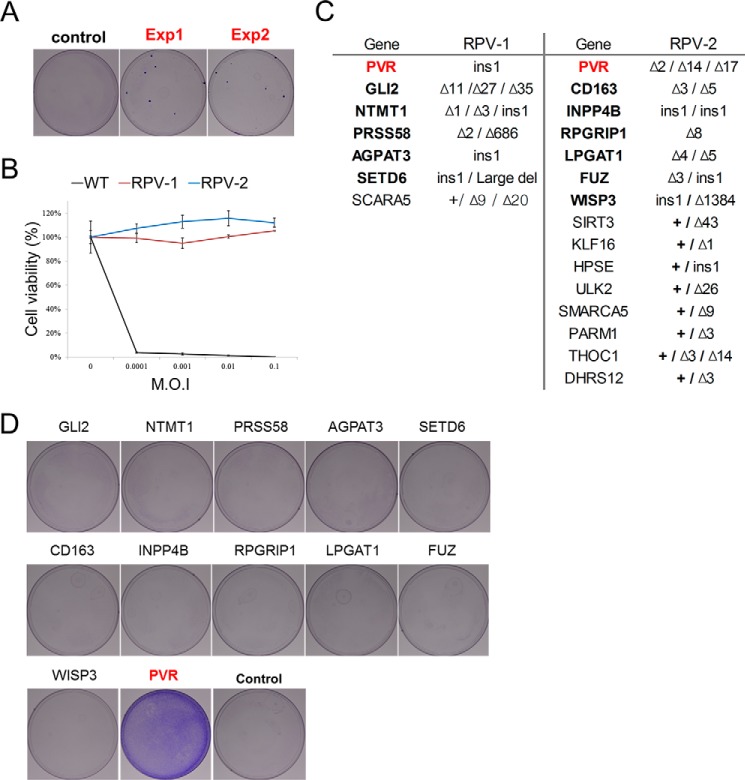
**Pooled sgRNA screens for poliovirus 1 resistance.** *A*, crystal violet staining of PV1-resistant cells after 12 days of infection. *B*, viability of WT HeLa cells and two colonies obtained from cells transfected with the library after reinfection with PV1. (*n* = 3, ± S.D.). *C*, list of genes in which mutations were observed at target sites in the two colonies that survived PV1 challenge. The gene encoding PVR is marked in *red*. Genes completely disrupted are denoted in *bold. D*, crystal violet staining of PV1-resistant cells after transfection of sgRNAs targeting the indicated genes. PV1-resistant colonies formed only after transfection of sgRNAs targeting the *PVR* gene. Other genes were also mutated in clones RPV-1 and RPV-2, but targeting these genes did not make HeLa cells resistant to PV1.

Encouraged by this proof-of-principle experiment, we performed pooled sgRNA screens to identify genes essential for viral infection or replication. We chose two cytopathic viruses, PV1 and EV-D68, for analysis in this study. HeLa cells were first transfected with the library of 30,840 plasmids and then subjected to PV1 infection. A few colonies survived among ∼1 million PV1-infected cells (Fig. 2*A*). We expanded two clones (termed RPV-1 and RPV-2) and confirmed that they were resistant to PV1 regardless of the viral titer using a cell viability assay (Fig. 2*B*).

To identify target genes disrupted in these two clones, we carried out WGS using genomic DNA isolated from these clones and investigated whether Cas9-induced mutations were present at any of the 30,840 target sites. By using the Integrative Genomics Viewer (16), which aligns paired-end sequence reads to the human reference genome, we were able to determine computationally whether mutations were induced at each of the 30,840 target sites (Fig. 2*C*). The two clones harbored small insertions or deletions in 7 and 15 genes, respectively (Fig. 2*C* and supplemental Table S2). All of these mutations occurred at on-target sites of each sgRNA. Their mutation patterns, analyzed by WGS, also revealed that Cas9-mediated cleavage occurred ∼3 bp upstream of the PAM, as shown previously (17). These multiple on-target mutation effects were derived from co-transfected sgRNA expression plasmids, implying that the phenotypes that can occur with combinatorial gene knockout events can be identified using this plasmid library system. Among these mutated genes, only one, which encoded poliovirus receptor (PVR), a well known receptor of PV1 infection, was completely disrupted in both of the clones (Fig. 2*C* and supplemental Fig. S1). To confirm that disruption of this gene alone was responsible for the resistance to PV1 infection, we separately transfected HeLa cells with sgRNAs specific to each of the 12 completely mutated genes and infected the cells with PV1. As expected, transfection of the PVR-targeted sgRNAs into HeLa cells gave rise to viral resistance. No virus-resistant colonies were obtained from cells transfected using sgRNAs targeted to the other genes (Fig. 2*D*). These results suggest that possible false-positive genes could be eliminated by screens using separate sgRNAs.

**Figure 3.**
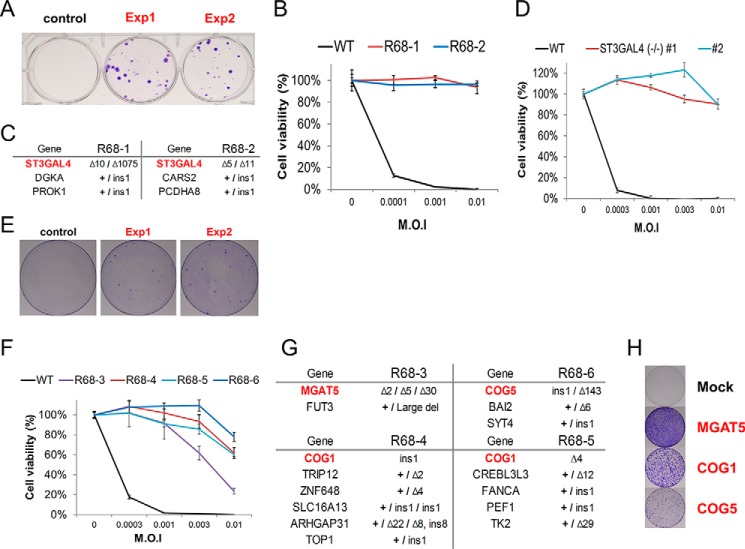
**Pooled sgRNA screens for enterovirus D68 resistance.** *A*, crystal violet staining of EV-D68-resistant cells after 12 days of infection. *B*, viability of WT HeLa cells and two colonies obtained from cells transfected with the library after reinfection with EV-D68 (*n* = 3, ± S.D.). *C*, list of genes that were mutated in clones R68-1 and R68-2. *D*, viability of WT and *ST3GAL4* knockout cells after EV-D68 infection. (*n* = 3, ± S.D.). *E*, colony formation after EV-D68 selection in cells transfected with the *ST3GAL4* dropout library. *F*, viability of EV-D68-resistant clones obtained from cells transfected with the *ST3GAL4* sgRNA dropout library (*n* = 3, ± S.D.). *G*, list of genes that were mutated in each clone. *H*, crystal violet staining of EV-D68-resistant cells after transfection with the indicated sgRNAs.

For the next screen, we infected HeLa cells transfected with the library with EV-D68 and obtained many resistant clones (Fig. 3*A*). We expanded two clones (R68-1 and R68-2) and confirmed that they were resistant to EV-D68 infection (Fig. 3*B*). WGS showed that one gene encoding ST3 β-galactoside α-2,3-sialyltransferase 4 (*ST3GAL4*) was completely disrupted in the two clones (Fig. 3*C* and supplemental Figs. S2 and S3). Transfection of each sgRNA targeted to the *ST3GAL4* gene into HeLa cells gave rise to numerous virus-resistant colonies (supplemental Fig. S4). Furthermore, *ST3GAL4* knockout cells created by using two sgRNAs targeted to different sites in the gene were also resistant to EV-D68 infection (Fig. 3*D*). Taken together, these data show that the gene identified by WGS of resistant clones is an essential factor for EV-D68 infection or replication.

To identify other genes essential for EV-D68 infection, we first picked several additional resistant colonies and checked whether these clones also had mutations at the *ST3GAL4* target sites. All of the clones we analyzed had insertions or deletions in the *ST3GAL4* gene. We hypothesized that *ST3GAL4*-disrupted cells had a selective advantage over other gene-disrupted cells in the presence of the enterovirus and that other genes essential for the viral infection could be identified using a dropout library in which the three sgRNAs specific to *ST3GAL4* were excluded. Indeed, we were able to obtain many resistant colonies from additional screening using the *ST3GAL4* dropout library (Fig. 3*E*). Interestingly, unlike the *ST3GAL4* KO clones, four clones (R68-3 to R68-6) isolated from cells transfected with the dropout library were partially resistant to EV-D68 (Fig. 3*F*). WGS showed that these clones did not harbor mutations in the *ST3GAL4* gene. Instead, two to six other genes were mutated in each of these clones. Only a single gene was completely disrupted in each clone. The *COG1* gene, which encodes a component of oligomeric Golgi complex 1, was disrupted in two clones. The other two clones harbored triallelic mutations in the *MGAT5* gene, which encodes mannosyl (α-1,6-)-glycoprotein β-1,6-*N*-acetyl-glucosaminyltransferase, or biallelic mutations in the *COG5* gene, which encodes a component of oligomeric Golgi complex 5 (Fig. 3*G* and supplemental Figs. S5 and S6). All of the other mutated genes were accompanied by a wild-type allele. Transfection of sgRNAs specific to each of the three completely disrupted genes into HeLa cells gave rise to EV-D68-resistant colonies (Fig. 3*H*).

### Genes related to sialic acid presentation are essential for enterovirus D68 infection

**Figure 4.**
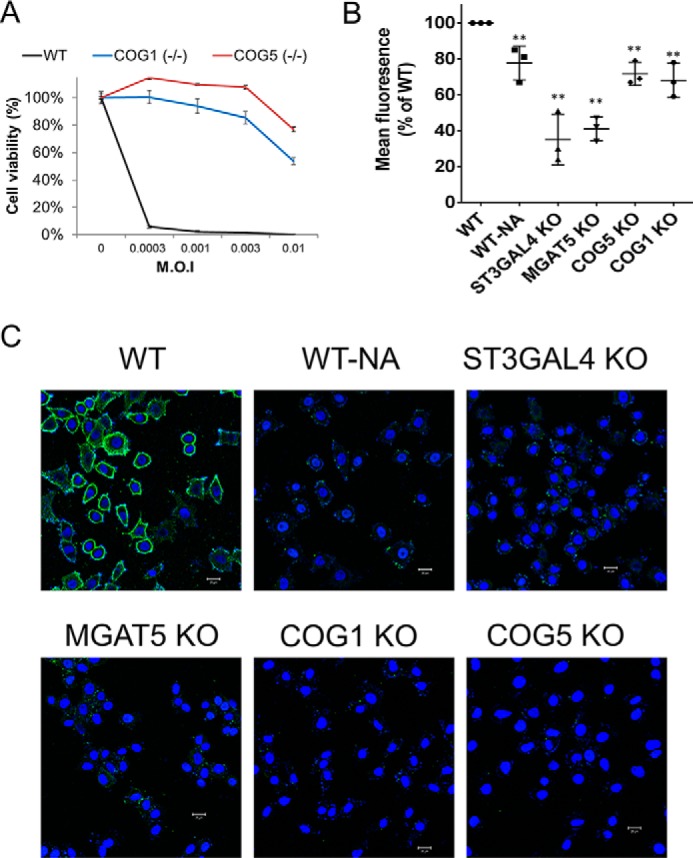
**Genes related to sialic acid presentation play an important role in enterovirus D68 infection.** *A*, viability of WT and *COG1* and *COG5* knockout cells after EV-D68 infection (*n* = 3, ± S.D.). *B*, quantification of 2,3-linked sialic acid presentation by flow cytometry. Cells were analyzed after incubation with fluorescein-labeled MALI. **, *p* < 0.01 (*n* = 3, ± S.D.). *C*, confocal microscopy of knockout cells and WT cells (including WT-NA cells treated with neuraminidase) immunostained with anti-VP1 antibody after incubation with EV-D68 on ice for 60 min. EV-D68 was visualized with Alexa Fluor 488-conjugated secondary antibody (*green*), and cell nuclei were stained with DAPI (*blue*). *Scale bars* = 20 μm.

To understand the mechanism behind the viral resistance, we characterized these genes using KO cells. Recently, Baggen *et al.* (18) showed that EV-D68 entry into cells is dependent on cell-surface sialic acid and identified the *ST3GAL4* and *MGAT5* genes as essential for EV-D68 infection via their role in sialic acid conjugation in the Golgi. We hypothesized that the *COG1* and *COG5* genes, the other two genes we identified in this study, might also be associated with sialic acid conjugation because the conserved oligomeric Golgi (COG) complex has been proposed to act in the glycosylation process in cells (19). Indeed, *COG1* and *COG5* KO clones were resistant to EV-D68 infection (Fig. 4*A*). To analyze surface sialic acid presentation by flow cytometry, those KO cells were stained with fluorescein-labeled *Maackia amurensis* lectin I (MALI), which binds selectively to 2,3-linked sialic acid. HeLa cells treated with neuraminidase (Fig. 4, *WT-NA*), which removes sialic acid residues from glycoproteins on the cell surface, were used as a control. The *ST3GAL4*, *MGAT5*, *COG1*, and *COG5* KO cells all showed a reduction in the level of 2,3-linked sialic acid on their surfaces (Fig. 4*B*). We then incubated these KO cells with EV-D68 virus particles at 4 °C and stained them with anti-EV-D68 VP1. Unlike HeLa cells, to which virus particles were well attached, all of the KO clones were poorly associated with EV-D68 on their cell surfaces (Fig. 4*C*). These results show that *COG1* and *COG5* play an important role in presenting sialic acid on the cell surface and that disruption of these genes leads to resistance to EV-D68 entry into cells.

### Sialic acid transferases, which mainly present sialic acids, are essential for EV-D68 infection

**Figure 5.**
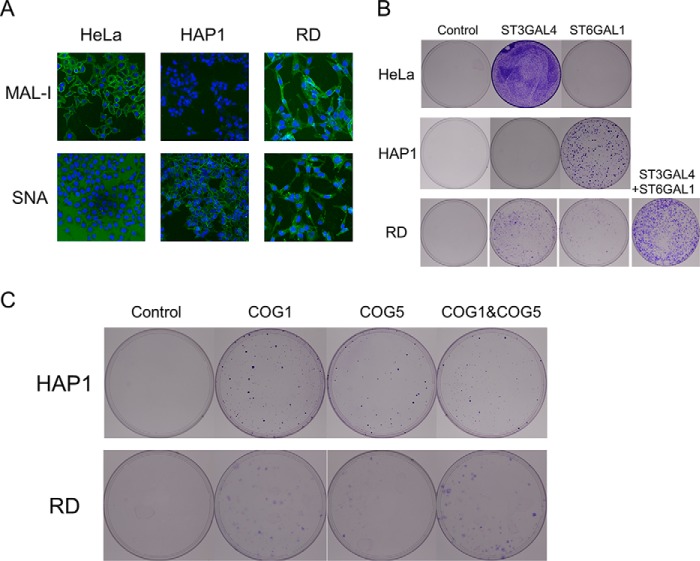
**Sialic acid transferase, essential for viral infection, is dependent on the sialic acid presentation pattern of each cell line.** *A*, lectin staining of HAP-1, HeLa, and RD cells. HAP-1 cells displayed strong 2,6-linked sialic acid presentation as shown by staining with *S. nigra* lectin, whereas HeLa cells exhibited 2,3-linked sialic acid as shown by straining with MAL-I. RD cells showed strong presentation of both sialic acids. *B* and *C*, each cell line was transfected with the indicated gene-targeting sgRNA and infected by EV-D68. Surviving cells are shown stained.

Although sialic acid presentation is important in EV-D68 infections, a previous gene trap showed that *ST6GAL1*, which mainly catalyzes 2,6-linked sialic acid formation, is considerably more important than *ST3GAL4* in HAP-1 cells for viral infection (18). However, we could only identify *ST3GAL4*, not *ST6GAL1*, as an essential factor for viral infection in HeLa cells. To investigate this, we stained HAP-1 and HeLa cells with MALI and *Sambucus nigra* lectin, which bind to 2,3-linked and 2,6-linked sialic acid, respectively. HeLa cells contained many 2,3-linked sialic acid molecules on their surface, but almost no 2,6-linked sialic acid was observed. However, HAP-1 cells showed an opposite pattern (Fig. 5*A*). From these contrasting results, we hypothesized that sialic acid transferase, which mainly presents sialic acid, would be a key factor for viral infection. To confirm this, we transfected sgRNAs targeting *ST3GAL4* and *ST6GAL1* into HeLa cells and then infected the cells with EV-D68. Only sgRNAs targeting *ST3GAL4* conferred resistance to HeLa cells. We were unable to isolate any resistant colonies from *ST6GAL1* sgRNA-transfected cells (Fig. 5*B*). In contrast, when we transfected the same sgRNAs to HAP-1 cells, *ST6GAL1*-targeting sgRNAs conferred resistance, as shown previously (Fig. 5*B*). In RD cells, which have been most frequently used for EV-D68 infections, both sialic acids were presented well (Fig. 5*A*). Unlike in HeLa and HAP-1 cells, sgRNAs targeting both *ST3GAL4* and *ST6GAL1* induced EV-D68 resistance in RD cells. Furthermore, co-transfection of *ST3GAL4* and *ST6GAL1* targeting sgRNAs showed more resistant clones, suggesting that both sialyltransferases are important (Fig. 5*B*). From these results, we could infer that sialic acid transferases, which mainly present sialic acid, are essential host factors for EV-D68 infection. As each cell type had a different dependence on sialic acid transferases, we tested whether *COG1* and *COG5* were required for EV-D68 infection in various cells. HAP-1 and RD cells were transfected with the Cas9 expression plasmid and plasmids expressing sgRNAs targeting *COG1* and *COG5*. These cells were then infected with EV-D68. Both sgRNAs targeting *COG1* and *COG5* induced EV-D68 resistance in HAP1 and RD cells (Fig. 5*C*). These results, combined with the resistance results obtained with HeLa cells presented in Fig. 3*H*, suggest that *COG1* and *COG5* are host factors that act on broad cell types.

## Discussion

In this study, we provided proof-of-principle cases of genome-scale gene knockout screens implemented with WGS to identify causal mutations. We showed that genes essential for drug cytotoxicity or viral infection could be identified by isolating cells that survived the drug or virus challenge, respectively. Unlike siRNA or shRNA, sgRNA coupled with Cas9 leaves footprints at the target sites. WGS of cells that survived a viral challenge led to the identification of causative mutations in a target gene. Many genetic screens have been performed using arrayed RNAi libraries. However, these have recently been criticized for their lack of reproducibility derived from incomplete knockdown and high off-target effects of RNAi. In contrast, CRISPR/Cas9 completely disrupts genes, and the Cas9-sgRNA complex can discriminate 1- to 2-bp mismatches in the genome against RNAi, in which partial matching of the seed region is sufficient for knockdown. Moreover, we tried to reduce off-target events even further by selecting target sites that were different from any other sites in the human genome by at least three nucleotides and by adding extra guanines (12, 15). These aspects render this CRISPR screening approach more reproducible. In our results, as shown in Fig. 2, WGS showed that 20 genes, in addition to *PVR*, were mutated. In a sequence analysis, these mutations had occurred at on-target sites of each sgRNA and not from off-target events. These might be introduced by transfection of multiple sgRNA plasmids into individual cells. Among the 20 genes mutated, 12 genes that were completely disrupted were tested using separate sgRNAs, and none was related to viral resistance except for PVR, as shown in Fig. 2*D*. Although 11 genes other than PVR among the completely disrupted 12 genes could be false positives in our screen, possible false positives were efficiently eliminated by using this separate sgRNA test.

In general, antiviral drugs target the virus itself. However, drug-resistant viruses have been reported that do not respond to drugs by mutating the target site. To overcome this limitation, key host factors could be good targets because of the low possibility of developing resistant mutations. It is also important that host factors are selectively essential for viral infection but not cell growth. Our screening system is highly suitable to identify such novel targets for therapeutics, as we isolated the resistant cells after gene knockout and virus challenge. Cells could grow and survive even after host factors are completely lacking. We show here that four host factors for EV-D68 infection, *ST3GAL4*, *MGAT5*, *COG1*, and *COG5,* are potential antiviral targets. These four proteins were involved in sialic acid presentation on the cell surface (Fig. 4*B*) and EV-D68 binding (Fig. 4*C*). *ST3GAL4* and *MGAT5* were reported previously to be essential factors for EV-D68 by haploid genetics (18). *COG1* and *COG5* are novel host factors for EV-D68, to the best of our knowledge. These two proteins are components of the COG complex required for normal Golgi morphology and function (19, 20). CHO cells with mutations in the COG complex show defects in the Golgi-associated glycosylation reaction, similar to that observed in our COG1 and COG5 knockout cells. We also found that these proteins were required in various cells, including HeLa, HAP-1, and RD cells. Interestingly, defects in COG genes were identified in patients with a mild form of congenital disorder involving glycosylation type II (CDG-II) (20). Sadat et al. (21) reported that patients with congenital disorder involving glycosylation type IIb showed impaired viral replication and cellular entry. These data imply that acute viral infection using the sialic acid receptor could be inhibited by lowering the activity of the COG complex transiently because the COG complex is a valuable target for antiviral agents against EV-D68 or influenza virus.

Many studies have shown that lentiviral-based libraries encoding sgRNAs enable forward genetic screens in human and other mammalian cells. Unlike these approaches, which count the number of sgRNA sequences before and after selection by PCR and deep sequencing to identify target genes indirectly, our method relies on WGS to identify causal mutations directly in cells after selection. WGS might be considered cumbersome in terms of cost and technical accessibility. However, as sequencing technology further developed recently, WGS became quite achievable through HiSeq X Ten, with prices similar to those of the MiSeq or HiSeq platforms normally used for lentivirus-based library screening approaches.

Transient transfection of sgRNA and Cas9 plasmids into human cells gives rise to high-level expression of these components, resulting in efficient disruption of the target genes. In particular, for cells that are non-immortalized or for pluripotent stem cells, which are easily differentiated, the 2–3 weeks required to disrupt genes by lentiviral sgRNA expression could be cumbersome for screening in contrast to the ∼3 days required with transient plasmid expression. Although there are cell lines in which transfection with cationic lipids such as Lipofectamine is not efficient, alternative methods, including electroporation, could be a solution. In addition, small populations resulting from low transfection efficiency could be selected over non-transfected cells because of our stringent selection process. Furthermore, as shown in Fig. 2*C*, multiple gene knockouts can be generated in individual cells, demonstrating new possibilities for discovery of novel genes that function in combination. As shown in Fig. 3*E*, the dropout library was easily constructed by using individually synthesized libraries rather than employing the *in situ* synthesized libraries on microarrays used for lentivirus-based screening. Dominant sgRNA targeting *ST3GAL4*, as presented in Fig. 3*A*, was excluded, and successful screens to find other host factors for virus were achieved.

Despite the potential utility of this method, there is the limitation that genes exerting mild phenotypes could be missed due to the stringent selection performed in this experiment in comparison with lentivirus-based screening. This method is unlikely to replace current methods using lentiviral sgRNA libraries in many settings but will still provide a new option for researchers, especially when lentiviral production and infection are inefficient or not feasible. We propose that our pooled sgRNA screening method and resources will be broadly useful for forward genetic studies and drug target discovery.

## Experimental procedures

### sgRNA oligonucleotide preparation

Oligonucleotides were purchased from Bioneer. In each well of a 96-well plate, two complementary 24-nt oligonucleotides were mixed at 100 μm in a total volume of 100 μl. Each oligonucleotide pair was diluted to 50 μm in TES buffer (1 mm EDTA, 10 mm Tris-HCl (pH 7.5), and 100 mm NaCl) and annealed to form a duplex by heating to 80 °C and cooling to room temperature in a water bath.

### sgRNA library construction

An empty sgRNA expression vector cleaved using BsaI (New England Biolabs) was ligated with annealed oligonucleotide mixtures. In this vector, sgRNA is transcribed under the control of the U6 promoter; the sequence is 5′-NNNNNNNNNNNNNNNNNNNNGTTTTAGAGCTAGAAATAGCAAGTTAAAATAAGGCTAGTCCGTTATCAACTTGAAAAAGTGGCACCGAGTCGGTGCTTTTT-3′. Ligation products were electroporated into DH5α-E-competent cells (Invitrogen). Transformed *Escherichia coli* cells were plated on a 24-cm Luria-Bertani (LB) plate containing ampicillin. Plasmid DNA was purified using the Nucleobond Xtra Midi EF purification system (Macherey-Nagel).

### Cell culture and transfection conditions

HeLa (ATCC, CCL-2) and RD (ATCC, CCL-136) cells were maintained in DMEM supplemented with 100 units/ml penicillin, 100 μg/ml streptomycin, 0.1 mm non-essential amino acids, and 10% FBS. 2 × 10^6^ HeLa cells were plated 1 day before transfection. The Cas9 expression plasmid (15 μg) and sgRNA library plasmids (15 μg) were transfected using Lipofectamine 2000 (Invitrogen) according to the protocol of the manufacturer. Genomic DNA was isolated 72 h post-transfection. Single-cell derived knockout cell lines were obtained by limiting dilution. HAP1 cells were purchased from Haplogen and maintained in Iscove's modified Dulbecco's medium supplemented with 10% FBS.

### Viruses

Poliovirus type 1 (Strain: Chat, ATCC, VR-1562) was expanded by growth in HeLa cells and titered using HeLa cells. Enterovirus 68 (Fermon, ATCC, VR-561) was expanded and titered in RD cells. For the virus binding assay, EV-D68 was concentrated by ultracentrifugation.

### Virus infection and screening

Cells that had been transfected 6 days previously with the Cas9 plasmid and sgRNA library were reseeded and infected with poliovirus 1 at an m.o.i. of 0.01 at 37 °C for 1 h. Cells were infected with enterovirus D68 in the same manner, but the incubation was performed at 33 °C. Infected cells were washed and resuspended in complete growth medium. The culture medium was changed every 2 or 3 days. After 12 days, surviving colonies were fixed and stained with crystal violet (0.05%) in PBS solution containing 1% formaldehyde and 25% methanol or isolated and expanded for further analysis.

### Whole-genome sequencing

Genomic DNA was purified with a DNeasy Tissue kit (Qiagen) according to the instructions of the manufacturer. 1 μg of genomic DNA was fragmented using the Covaris system (Life Technologies) and polished to generate blunt ends using end repair mixture. Fragmented DNA was ligated with adaptors to produce libraries and subjected to WGS using an Illumina HiSeq X Ten Sequencer at Macrogen. Mapping programs and parameters were as described previously (14).

### Reinfection test

Surviving cells were plated in 96-well plates at 2 × 10^4^ cells/well to test whether surviving cells were resistant to viral infection. For the reinfection assay, 10-fold diluted poliovirus 1 (m.o.i. 0.1–0.0001) was added to each well, and the cultures were incubated at 37 °C for 2 days. For enterovirus D68, 3- or 10-fold diluted virus was added to each well, and the cultures were incubated at 33 °C for 3 days. To measure cell viability after virus infection, a modified 3-(4,5-dimethylthiazol-2-yl)-2,5-diphenyltetrazolium bromide (Sigma) assay was performed as described previously (22). The viability of infected cells was normalized to the viability of mock-infected cells (expressed as 100%).

### Lectin staining

For measuring the level of 2,3-linked sialic acid on the cell surface, cells were detached from plates using trypsin and stained with fluorescein-labeled MALI (Vector Laboratories). As a control, HeLa cells were treated with 100 milliunits/ml neuraminidase from *Clostridium perfringens* (Sigma-Aldrich) as described previously (10). Flow cytometry was performed using a BD Accuri flow cytometer (BD Biosciences), and the data were analyzed using the FlowJo program.

### Virus binding assay

3 × 10^4^ cells were plated on an 8-chamber slide (SPL Lifesciences) and incubated overnight. The next day, cells were treated with neuraminidase for 1 h at 37 °C. Cells were then incubated on ice for 15 min, after which EV-D68 (m.o.i. 50) was added to the cells. After incubation on ice for 1 h, the cells were washed three times with cold PBS and then fixed with 4% paraformaldehyde. EV-D68 was stained with anti-enterovirus D68 VP1 antibody (Genetex, 132313) and Alexa Fluor 488-conjugated goat anti-rabbit IgG antibody (Life Technologies, A11008). After counterstaining with Vectashield mounting medium with DAPI (Vector Laboratories), cells were visualized using confocal microscopy (Zeiss LSM 710) and ZEN software.

## Author contributions

H. S. K., K. L., C. K. L., Meehyein Kim, E. K., Minju Kim, S. K., and C. K. performed the experiments. H. S. K., S. B., and J. P. performed the bioinformatics analyses. J. S. K. and C. K. supervised the research.

## Supplementary Material

Supplemental Data

## Footnotes

5 Please note that the JBC is not responsible for the long-term archiving and maintenance of this site or any other third party-hosted site.

## Footnotes

4 The abbreviations used are:

sgRNA: small guide RNA
CRISPR: clustered regularly interspaced short palindromic repeats
Cas: CRISPR-associated
WGS: whole-genome sequencing
PV: poliovirus
EV: enterovirus
6-TG: 6-thioguanine
PVR: poliovirus receptor
COG: conserved oligomeric Golgi
MALI: *Maackia amurensis* lectin I
m.o.i.: multiplicity of infection
RD: rhabdomyosarcoma.
