# Supplementary material for: CRISPR/Cas9-mediated gene knockout screens and target identification via whole-genome sequencing uncover host genes required for picornavirus infection
Source: J Biol Chem. 2017 Apr 26;292(25):10664–71. doi: 10.1074/jbc.M117.782425 (PMC5481571; doi:10.1074/jbc.M117.782425)
Supplement: Supplemental Data [file supp_292_25_10664__index.html]

CRISPR/Cas9-mediated Gene-knockout Screens and Target Identification via Whole Genome Sequencing Uncover Host Genes Required for Picornavirus Infection — CRISPR/Cas9-mediated gene knockout screens and target identification via whole-genome sequencing uncover host genes required for picornavirus infection — Novel CRISPR gene knockout screens via WGS — Supplemental Data 

# CRISPR/Cas9-mediated gene knockout screens and target identification via whole-genome sequencing uncover host genes required for picornavirus infection

## Supplemental Data

- Supplemental Table 1 (.xlsx, 951 KB) - List of sgRNA sequence
- Supplemental data (.pdf, 1.1 MB) - Supplemental Figure S1-S6
- Supplemental Table S2 (.xlsx, 15 KB) - Sequence of RPV-1, 2 clones
