# Supplementary material for: CRISPR/Cas9-mediated gene knockout screens and target identification via whole-genome sequencing uncover host genes required for picornavirus infection
Source: J Biol Chem. 2017 Apr 26;292(25):10664–71. doi: 10.1074/jbc.M117.782425 (PMC5481571; doi:10.1074/jbc.M117.782425)
Supplement: Supplemental Data [file 10.1074_M117.782425_jbc.M117.782425-2.pdf]

CRISPR/Cas9-mediated Gene-knockout Screens and Target Identification via Whole Genome Sequencing Uncover Host Genes Required for Picornavirus Infection

Heon Seok Kim, Kyungjin Lee, Sangsu Bae, Jeongbin Park, Chong-Kyo Lee, Meehyein Kim, Eunji Kim, Minju Kim, Seokjoong Kim, Chonsaeng Kim and Jin-Soo Kim

Supplementary Figure S1. IGV view of the *PVR* sgRNA target site in the RPV-1 and RPV-2 clones. The WGS data shows that both clones have mutations at the *PVR* target site: wild-type and mutant sequences are presented. Small indels were verified using IGV. The genomic location is chr19:45,150,636-45,150,704. The PAM sequences are indicated in red, and an insertion is indicated in blue.

Supplementary Figure S2. IGV view of the *ST3GAL4* sgRNA target site in the R68-1 and R68-2 clones. The WGS data reveal that both clones have mutations at the *ST3GAL4* target site. The genomic location is chr11:126,277,962-126,278,040. The PAM sequences are indicated in red, and an insertion is indicated in blue.

Supplementary Figure S3. Sequences of the sites containing sgRNA library-induced mutations in the R68-1 and R68-2 clones. IGV was used for the analysis. The PAM sequences are indicated in red, and the insertion sequences are indicated in blue. + indicates wild type.

Supplementary Figure S4. EV-D68-resistant colonies formed after transfection of sgRNAs targeting the *ST3GAL4* gene.

Supplementary Figure S5. IGV view of the genes that were knocked-out in clones R68-3, 4, 5, and R68-6. R68-3; MGAT5, chr2:135,012,121-135,012,263. R66-4, 5; COG1, chr17:71,189,364-71,189,404. R68-6; COG5, chr7:107,204,316-107,204,356. The PAM sequences are indicated in red, and the insertion sequences are indicated in blue.

Supplementary Figure S6. Sequences of the sites containing sgRNA library-induced mutations in the R68-3, 4, 5, and R68-6 clones. IGV was used for the analysis. The PAM sequences are indicated in red, and the insertion sequences are indicated in blue. + indicates wild type.

Supplementary Table S1. List of sgRNA sequence

Supplementary Table S2. Sequence of RPV-1, 2 clones

Supplementary Figure S1. IGV view of the *PVR* sgRNA target site in the RPV-1 and RPV-2 clones. The WGS data shows that both clones have mutations at the *PVR* target site: wild-type and mutant sequences are presented. Small indels were verified using IGV. The genomic location is chr19:45,150,636-45,150,704. The PAM sequences are indicated in red, and an insertion is indicated in blue.

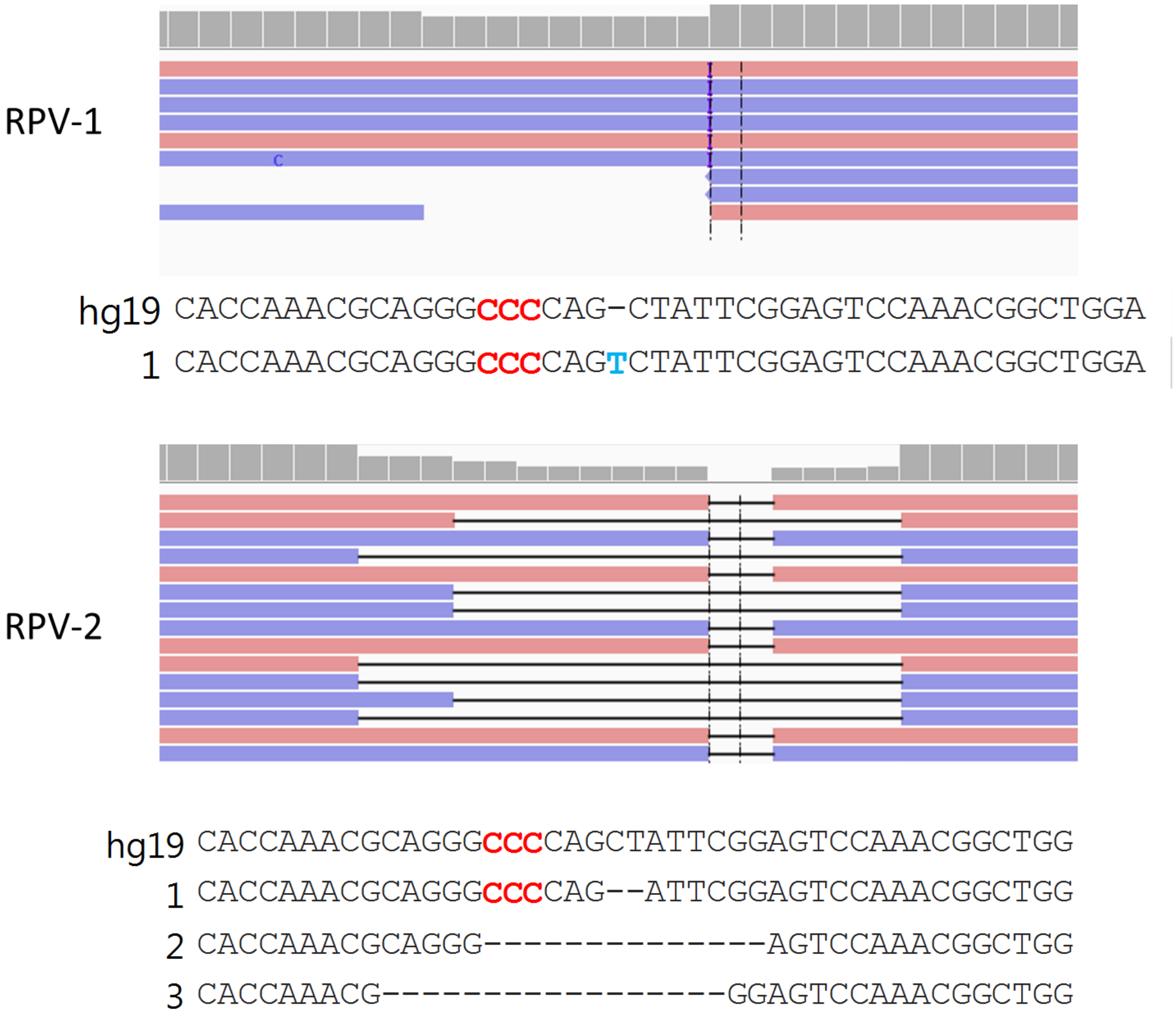

Supplementary Figure S2. IGV view of the *ST3GAL4* sgRNA target site in the R68-1 and R68-2 clones. The WGS data reveal that both clones have mutations at the *ST3GAL4* target site. The genomic location is chr11:126,277,962-126,278,040. The PAM sequences are indicated in red, and an insertion is indicated in blue.

R68-1

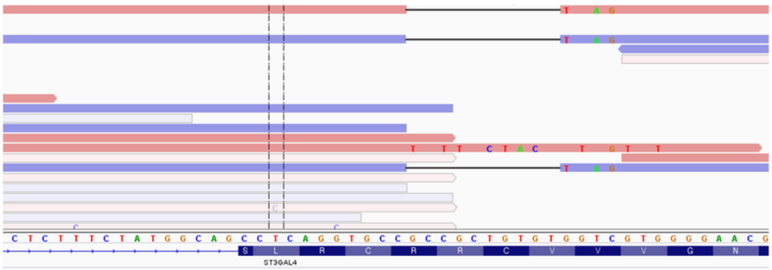

6484

hg19 GCAGCCTCAGGTGCCGCCGCTGTGTGGTCGTGGGGAACGGGCACCGGCTGCGGAACAGCT
1 GCAGCCTCAGGTGCCTGAG----------GTGGGGAACGGGCACCGGCTGCGGAACAGCT 10del
2 GCAGCCTCAGGTGCCGCC-- 1015del

R68-2

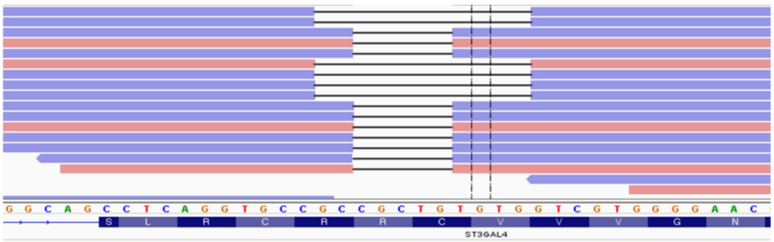

6484

hg19 GACCACTCCTCTTTCTATGGCAGCCTCAGGTGCCGCCGCTGTGTGGTCGTGGGGAACGGG
1 GACCACTCCTCTTTCTATGGCAGCCTCAGGTGCCGC-----TGTGGTCGTGGGGAACGGG 5del
2 GACCACTCCTCTTTCTATGGCAGCCTCAGGTGCCG-----------TCGTGGGGAACGGG 11del

Supplementary Figure S3. Sequences of the sites containing sgRNA library-induced mutations in the R68-1 and R68-2 clones. IGV was used for the analysis. The PAM sequences are indicated in red, and the insertion sequences are indicated in blue. + indicates wild type.

```
R68-1
6484
hg19 GCAGCCTCAGGTGCCGCCGCTGTGTGGTCGTGGGGAACGGGCACCGGCTGCGGAACAGCT
   1 GCAGCCTCAGGTGCCTGAG----------GTGGGGAACGGGCACCGGCTGCGGAACAGCT   10del
   2 GCAGCCTCAGGTGCCGCC------------------------------------------   1015del
1606
hg19 GCCTTGTGAAGTCAGCACCTATGCCAAGTCTCGGAAGGA-CATTGGTGTGAGTGATCTCA
   1 GCCTTGTGAAGTCAGCACCTATGCCAAGTCTCGGAAGGA-CATTGGTGTGAGTGATCTCA   +
   2 GCCTTGTGAAGTCAGCACCTATGCCAAGTCTCGGAAGGATCATTGGTGTGAGTGATCTCA   1 ins
84432
hg19 TACAGGCCTGTG-AGCGGGATGTCCAGTGTGGGGCAGGCACCTGCTGTGCCATCAGCCTG
   1 TACAGGCCTGTG-AGCGGGATGTCCAGTGTGGGGCAGGCACCTGCTGTGCCATCAGCCTG   +
   2 TACAGGCCTGTGAAGCGGGATGTCCAGTGTGGGGCAGGCACCTGCTGTGCCATCAGCCTG   1 ins

R68-2
6484
hg19 GACCACTCCTCTTTCTATGGCAGCCTCAGGTGCCGCCGCTGTGTGGTCGTGGGGAACGGG
   1 GACCACTCCTCTTTCTATGGCAGCCTCAGGTGCCGC-----TGTGGTCGTGGGGAACGGG   5del
   2 GACCACTCCTCTTTCTATGGCAGCCTCAGGTGCCG-----------TCGTGGGGAACGGG   11del
79587
hg19 ATGAAAGAAATTATCTGAGGAATATTTTCGGTTACCCTCAGGTACA-CCGTGGGTGGGA
   1 ATGAAAGAAATTATCTGAGGAATATTTTCGGTTACCCTCAGGTACA-CCGTGGGTGGGA   +
   2 ATGAAAGAAATTATCTGAGGAATATTTTCGGTTACCCTCAGGTACAACCGTGGGTGGGA   1 ins
56140
hg19 TTCCATGTGGACGTGGAGGTGAAGGATGTTAATGACAACCCGCC-AGTGTTCCGG
   1 TTCCATGTGGACGTGGAGGTGAAGGATGTTAATGACAACCCGCC-AGTGTTCCGG   +
   2 TTCCATGTGGACGTGGAGGTGAAGGATGTTAATGACAACCCGCCAAGTGTTCCGG   1 ins
```

Supplementary Figure S4. EV-D68-resistant colonies formed after transfection of sgRNAs targeting the *ST3GAL4* gene.

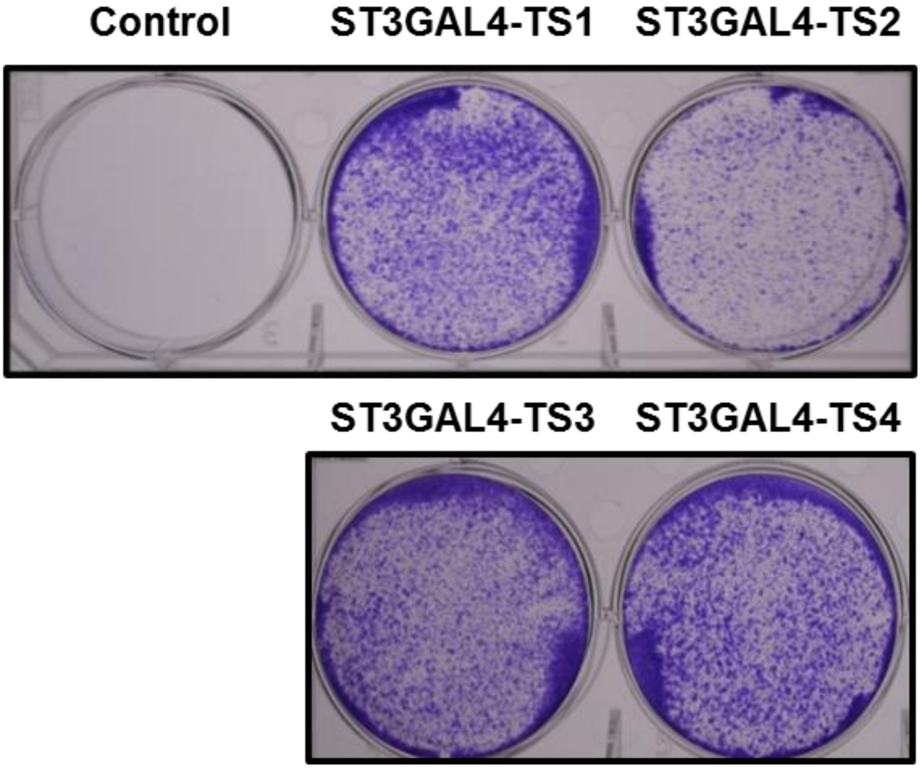

Supplementary Figure S5. IGV view of the genes that were knocked-out in clones R68-3, 4, 5, and R68-6. R68-3; MGAT5, chr2:135,012,121-135,012,263. R66-4, 5; COG1, chr17:71,189,364-71,189,404. R68-6; COG5, chr7:107,204,316-107,204,356. The PAM sequences are indicated in red, and the insertion sequences are indicated in blue.

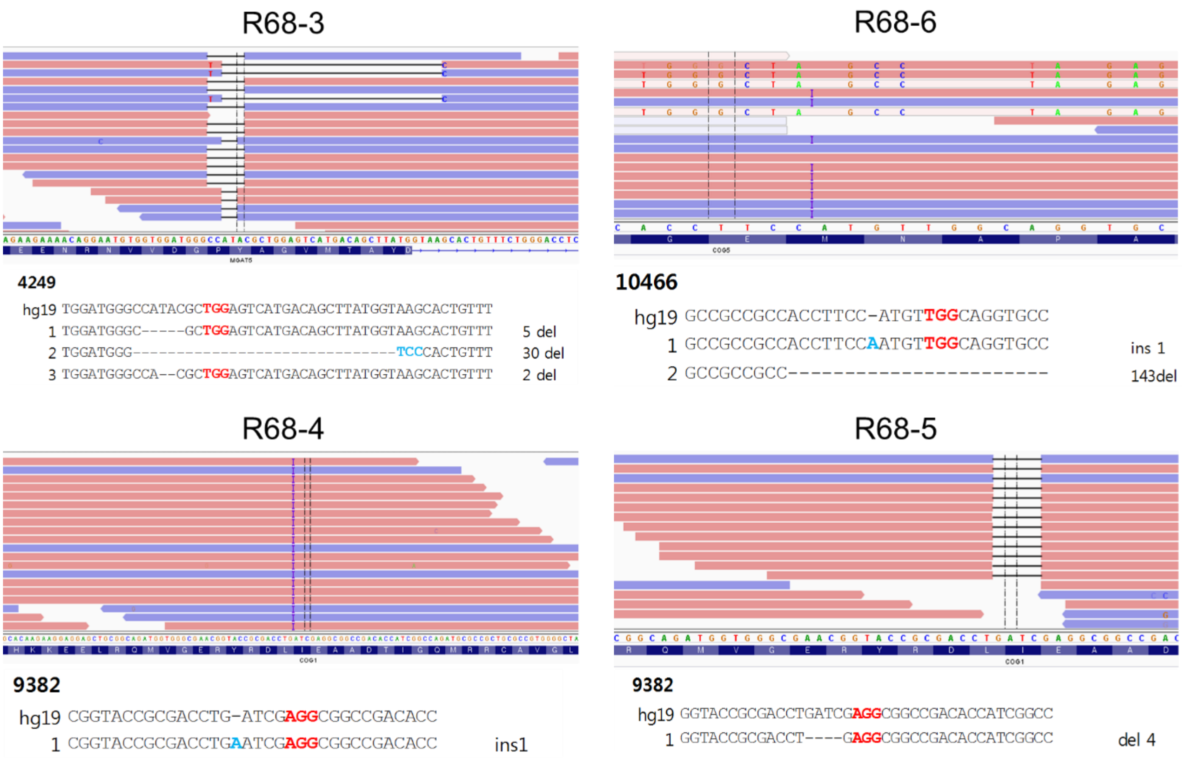

Supplementary Figure S6. Sequences of the sites containing sgRNA library-induced mutations in the R68-3, 4, 5, and R68-6 clones. IGV was used for the analysis. The PAM sequences are indicated in red, and the insertion sequences are indicated in blue. + indicates wild type.

R68-3

4249

```
hg19 GGGCCATACGCTGGAGTCATGACAGCTTATGGTAAGCAC
   1 GGGC-----GCTGGAGTCATGACAGCTTATGGTAAGCAC      5 del
   2 GGG------------------------------TCCCAC      30 del
   3 GGGCCA--CGCTGGAGTCATGACAGCTTATGGTAAGCAC      2 del
```

2525

```
hg19 GGGTGGGGCGGGTGGGAGTGGTGTCCTGTCGGGAGGACC
   1 GGGTGGGGCGGGTGGGAGTGGTGTCCTGTCGGGAGGACC      +
   2 -------------GGGAGTGGTGTCCTGTCGGGAGGACC      Large del
```

R68-6

10466

```
hg19 GCCGCCGCCACCTTCC-ATGTTGGCAGGTGCC
   1 GCCGCCGCCACCTTCCAATGTTGGCAGGTGCC      ins 1
   2 GCCGCCGCC-----------------------      143del
```

576

```
hg19 TGCGTGCCCGTGGCCTGGCACATGCGGAAGCGG
   1 TGCGTGCCCGTGGCCTGGCACATGCGGAAGCGG      +
   2 TGCGTGCCCGTGGCCTGGCAC------AAGCGG      6del
```

6860

```
hg19 CTGGCAAGCCACGG-GCTTCCTTGA
   1 CTGGCAAGCCACGG-GCTTCCTTGA      +
   2 CTGGCAAGCCACGGGGCTTCCTTGA      ins 1
```

R68-4

9382

```
hg19 CGGTACCGCGACCTG-ATCGAGGCGGCCGACACC
   1 CGGTACCGCGACCTGAATCGAGGCGGCCGACACC      ins1
```

9320

```
hg19 TTTTGAAGCCAGCTTGGTAGGTTTCGCAGATCT
   1 TTTTGAAGCCAGCTTGGTAGGTTTCGCAGATCT      +
   2 TTTTGAAGCCAGCTTC--AGGTTTCGCAGATCT      del 2
```

127665

```
hg19 GCCCGCCCCGCAGGCACCGGTAGGGA
   1 GCCCGCCCCGCAGGCACCGGTAGGGA      +
   2 GCCCGCCCCG----TCCCTGTAGGGA      del 4
```

201232

```
hg19 TCGCCTCCATAGGAA-TCGCGGTGCAGCA
   1 TCGCCTCCATAGGAA-TCGCGGTGCAGCA      +
   2 TCGCCTCCATAGGAAATCGCGGTGCAGCA      ins 1
   3 TCGCCTCCATAGGAATTCGCGGTGCAGCA      ins 1
```

57514

```
hg19 GGCGCTGAACATCTCCGAGCCCTTTGCGGTATCTGTG
   1 GGCGCTGAACATCTCCGAGCCCTTTGCGGTATCTGTG      +
   2 GGCGCTG----------------------TATCTGTG      del 22
   3 GGCGCTGAACATCTCCGATACTCCACCGGTATCTGTG      ins 8, del 8
```

7150

```
hg19 AAGATAGAGCCTCCTGGACTTTT-CCGTGGCCGCGG
   1 AAGATAGAGCCTCCTGGACTTTT-CCGTGGCCGCGG      +
   2 AAGATAGAGCCTCCTGGACTTTTTCCGTGGCCGCGG      ins 1
```

R68-5

9382

```
hg19 GGTACCGCGACCTGATCGAGGCGGCCGACACCATCGGCC
   1 GGTACCGCGACCT----GAGGCGGCCGACACCATCGGCC      del 4
```

84699

```
hg19 CAGGACGGCATCCTGAGACACGTGGAGCTGGGCGAGGG
   1 CAGGACGGCATCCTGAGACACGTGGAGCTGGGCGAGGG      +
   2 CAGGACGG------------CGTGGAGCTGGGCGAGGG      del 12
```

2175

```
hg19 CTGGAGCCGTGC-AGATCTGTCCCACGCTA
   1 CTGGAGCCGTGC-AGATCTGTCCCACGCTA      +
   2 CTGGAGCCGTGCAAGATCTGTCCCACGCTA      ins 1
```

553115

```
hg19 GGAGATATAGCCACTG-TGATCTGAGTCCACCG
   1 GGAGATATAGCCACTG-TGATCTGAGTCCACCG      +
   2 GGAGATATAGCCACTGTTGATCTGAGTCCACCG      ins 1
```

7084

```
hg19 GGCTGCCTTTGATGAGCCACTCCTCATGGAGATGGTGAA
   1 GGCTGCCTTTGATGAGCCACTCCTCATGGAGATGGTGAA      +
   2 GGCT-----------------------------GGTGAA      del 29
```
